# Error Ellipsoid Analysis for the Diameter Measurement of Cylindroid Components Using a Laser Radar Measurement System

**DOI:** 10.3390/s16050714

**Published:** 2016-05-19

**Authors:** Zhengchun Du, Zhaoyong Wu, Jianguo Yang

**Affiliations:** School of Mechanical Engineering, Shanghai Jiaotong University, Shanghai 200030, China; zcdu@sjtu.edu.cn (Z.D.); jgyang@sjtu.edu.cn (J.Y.)

**Keywords:** error ellipsoid, uncertainty model, point cloud, laser radar, 3D acquisition

## Abstract

The use of three-dimensional (3D) data in the industrial measurement field is becoming increasingly popular because of the rapid development of laser scanning techniques based on the time-of-flight principle. However, the accuracy and uncertainty of these types of measurement methods are seldom investigated. In this study, a mathematical uncertainty evaluation model for the diameter measurement of standard cylindroid components has been proposed and applied to a 3D laser radar measurement system (LRMS). First, a single-point error ellipsoid analysis for the LRMS was established. An error ellipsoid model and algorithm for diameter measurement of cylindroid components was then proposed based on the single-point error ellipsoid. Finally, four experiments were conducted using the LRMS to measure the diameter of a standard cylinder in the laboratory. The experimental results of the uncertainty evaluation consistently matched well with the predictions. The proposed uncertainty evaluation model for cylindrical diameters can provide a reliable method for actual measurements and support further accuracy improvement of the LRMS.

## 1. Introduction

3D laser scanning techniques have been widely used in the measurement field. Scanning an object from a far distance facilitates the acquisition of the profile of the object in the form of 3D data, namely the point cloud [1,2]. Thus, the use of laser-based measurement devices is a good solution because they are contactless systems that are very efficient for 3D reconstruction and for the measurement of architectural and engineering structures [3,4,5,6,7]. However, the point cloud data inevitably contains measurement errors due to factors related to the external environment and to the measurement system itself. Exhaustive analysis of the factors influencing the quality of terrestrial laser scanning points was conducted in [8,9]. The authors presented a full error propagation model for directly georeferenced terrestrial laser scanner networks. It was shown that the quality of a scan point is influenced by complex factors, including the instrument mechanisms, atmospheric conditions, object surface properties, scan geometry, and error sources fundamental to surveying. In [10], the researchers presented an experimental evaluation of positioning and orientation parameters of a laser plane scanner on the accuracy measurement results. From the determination of the experimental error results, they derived a global systematic error model, which can be used to correct each acquired point. A performance evaluation test for laser line scanners using a planar artifact is presented in [11]. The orientation parameters of the scanner, such as the scan depth and the scan angle, were the main only factors investigated. Both results from [10,11] rely on the experiment or test. In addition, investigations on the 2D laser scanners were also carried out. A detailed discussion about the deterministic error and random error of a 2D laser radar measurement system (LRMS) was published in [12]. The qualitative relationships between the error and the error sources, such as the scanning incidence angle and surface roughness, were determined. Ye and Borenstein [13] presented an elaborate characterization study of a 2D laser scanner in their paper. They found that a number of parameters including operation time, data transfer rate, target surface properties, as well as the incidence angle may potentially affect the sensing performance. Furthermore, based on their experimental results, they built a rough probabilistic range measurement model. Zheng *et al.* [14] performed a full qualitative analysis of influential factors on the measurement precision of a 3D laser scanner. They verified that the factors mainly originate from the measurement system itself, the reflectance of the measured surface, and the characteristics of the scanning environment such as temperature and air pressure. All of the above studies [12,13,14] provide insights into the factors influencing the accuracy of the data; however, their work can only justify the range performance of the sensor, and they neglected to further investigate the accuracy or uncertainty in real measurement applications. Park *et al.* [15] proposed a mathematical model of uncertainties in the spatial measurements of visual features using Kinect™ sensors, presenting a quantitative analysis for the utilization of Kinect™ sensors as 3D perception sensors. Nevertheless, the research findings only apply for the uncertainty evaluation of a single point. Chen *et al.* [16] proposed a method for evaluating the point cloud precision of static 3D laser scanning by computing the volume of a point cloud error ellipsoid. According to this study, the evaluation performance of this method is not affected by the surface characteristics; however, it is only suitable for evaluating the accuracy of the point cloud as a whole. When it comes to a geometric feature’s measurement, such as the cylindrical and conic surface, the application in such cases are not discussed. Bokhabrine *et al.* [17] developed a system based on two commercially available time-of-flight 3D laser scanners for the measurement of the diameters of hot cylindrical metallic shells. Similarly, Zhang *et al.* [18] proposed a measurement model based on a line laser scanner that aimed to obtain the diameter of cylindroid hot forgings. A measurement system for cylindroid components and simple analysis on the measurement results were published in [17,18] but were only based on experimental investigations.

This paper, on the basis of our previous research work [19], further presents a mathematical model for evaluating the diameter measurement uncertainty of cylindroid components based on the error ellipsoid theory. To verify these models, four experiments using the LRMS and a simulation based on the proposed model were carried out. The results demonstrated that the uncertainty model can provide a reliable uncertainty evaluation for cylindrical diameter measurement applications.

The rest of this paper is organized as follows: In Section 2, the LRMS is briefly introduced and a single-point error ellipsoid model is established; in Section 3, the critical point theory is proposed inspired by the RANSAC algorithm, and the model of diameter measurement uncertainty for cylindroid components is established on the basis of the chosen critical point; in Section 4, an experiment measuring the diameter of a cylinder is introduced. Thereafter, a comparative analysis is made of the actual experimental results and simulated results based on the proposed uncertainty model; finally, Section 5 summarizes the contribution of this work and explores the potential for future work.

## 2. Error Ellipsoid Analysis for the LRMS [17]

### 2.1. Brief Introduction to the LRMS

**Figure 1:**
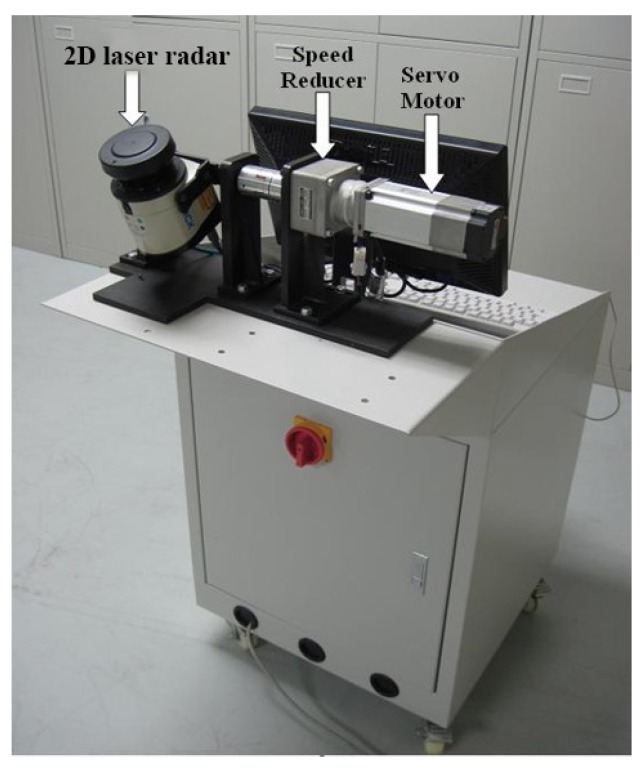
Laser radar measurement system (LRMS) configuration.

In general, for most laser-based measurement systems, the most convenient and effective choice of laser sensor may be a 3D laser scanner, which can obtain the point position in the spherical coordinate system. However, off-the-shelf 3D sensors such as the FARO Focus3D are usually too expensive. A cost-effective alternative for 3D data acquisition is to adopt 2D laser-based sensors with additional rotation or translation systems. The LRMS studied in this paper is such an example. It consists of a SICK LMS100 2D laser radar (Waldkirch, Germany), a PANASONIC MQMAP022P1 servomotor (Osaka, Japan), a NEDIC-SHIMPO VRSF-S9C-200 speed reducer (Kyoto, Japan), and a GT-400-SG motion control card produced by GOOGOLTECH (Shenzhen, China). As shown in Figure 1, the 2D laser radar is mounted on the extended shaft of the servomotor with the speed reducer in between.

**Figure 2:**
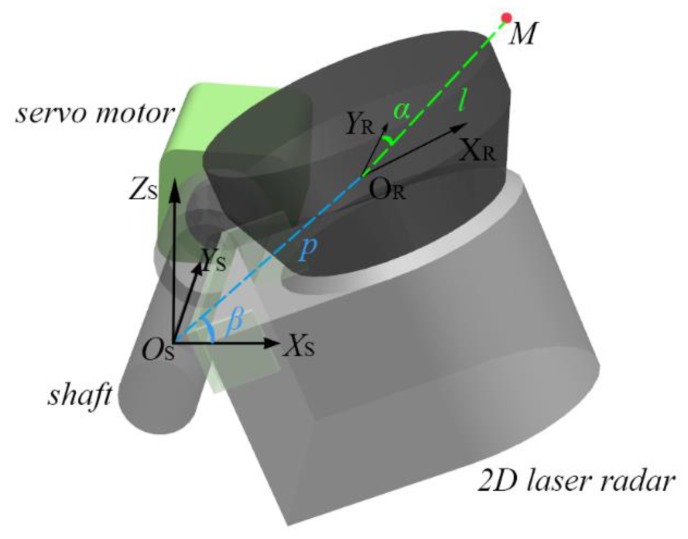
Mapping relationship between the reference coordinate system (RCS) and the radar measurement coordinate system (RMCS) ($O_{S}-X_{S}Y_{S}Z_{S}$: RCS; $O_{R}-X_{R}Y_{R}$: RMCS; M(l,α,β): a measured point M determined by *l*, *α*, and *β*; *p*: inner parameter of LRMS-distance between the radar’s optical center and motor rotation axis).

The LMS100 2D laser radar is a TOF-based range sensor, which has a detection angle of 270°, an angle resolution of 0.25°, and a detection range of 0.5 to 20 m [20]. According to the technical sheet of the laser radar, the reported point statistical error for the SICK LMS100 is typically 12 mm. The raw data acquired by the radar is on a single scanning plane, on which a radar measurement coordinate system (RMCS) can be established with the optical center as the origin. In order to describe the real position of the point cloud in the 3D Cartesian space, the raw data has to be mapped from the RMCS into a global reference coordinate system (RCS). Figure 2 shows the mapping relationship between the two coordinate systems.

As shown in Figure 2, the RCS is established on the extended motor shaft, which is along the y-axis direction. The z-axis is straight upwards, and the origin of the RMCS optical center is located on the $X_{S}O_{S}Z_{S}$ plane, which is always perpendicular to the radar scanning plane $X_{R}O_{R}Y_{R}$ during the motor rotation process. Thus, any measured point M in the RMCS can be uniquely determined by (*l*, *α*, *β*) where *l*, *α*, and *β* denote the range distance, the scanning angle, and the motor rotation angle, respectively. The mapping relationship between RMCS and RCS then allows point M in the RCS to be expressed as:

(1) $$\left\{ \begin{array}{l} x=l\sin\alpha \cos\beta +p\cos\beta \\ y=l\cos\alpha \\ z=l\sin\alpha \sin\beta +p\sin\beta \end{array}\right.$$

where *p* = 0.1 m is the inner parameter of the LRMS representing the distance between the optical center and the motor rotation axis. The real description of the point cloud in Cartesian space can now be obtained.

### 2.2. Single-Point Error Ellipsoid Model for the LRMS

The raw data suffers from interference to some degree because of the random error sources from the system setup and the laser radar itself. Consequently, the mapping positions in Cartesian space are distorted. One way to describe such random deviations of 3D point data is the error ellipsoid [10], which is based on probability distribution. For an arbitrary point in Cartesian 3D space, its spatial uncertainty information can be expressed as Equation (2):

(2) $$f(x,y,z)=\exp\left(-\frac{1}{2}r^{T}\sum_{x,y,z}{}^{-1}r\right)/{\left(2\pi \right)}^{3/2}\sqrt{\left|\sum_{x,y,z}\right|}$$

where $r={\left[x-x^{*},y-y^{*},z-z^{*}\right]}^{T}$. $\left(x^{*},y^{*},z^{*}\right)$ represents the real coordinates of the measured point, and $\sum_{x,y,z}$ is the covariance matrix containing the uncertainty information:

(3) $$\sum_{x,y,z}=\left[\begin{array}{l} \sigma_{x}{}^{2}\;\sigma_{xy}\;\sigma_{xz}\\ \sigma_{xy}\;\sigma_{y}{}^{2}\;\sigma_{yz}\\ \sigma_{xz}\;\sigma_{yz}\;\sigma_{z}{}^{2}\; \end{array}\right]$$

where $\sigma_{ij}\;(i,j=x,y,z)$ represents the covariance between two directions. Thus, the uncertainty ellipsoid model can be described as:

(4) $$r^{T}\sum_{x,y,z}{}^{-1}r=k^{2}$$

where *k* is the amplification coefficient. Specifically, if *k* = 1, then the error ellipsoid is called the uncertainty ellipsoid.

However, to derive the spatial uncertainty model for the LRMS, the mapping relationship between the RMCS and RCS must be considered since the covariance matrix $\sum_{x,y,z}$ is not immediately available. The uncertainty of all three parameters l,α,β of the raw data can be obtained either by theoretical analysis or by experimental investigation. Owing to the absence of correlations between these three parameters, the covariance matrix $\sum_{l,\alpha ,\beta }$ has a diagonal form:

(5) $$\sum_{l,\alpha ,\beta }=\left[\begin{array}{ccc} \begin{array}{l} \sigma_{l}{}^{2}\\ 0\\ 0 \end{array} & \begin{array}{l} 0\\ \sigma_{\alpha }{}^{2}\\ 0 \end{array} & \begin{array}{l} 0\\ 0\\ \sigma_{\beta }{}^{2} \end{array} \end{array}\right]$$

where the symbols $\sigma_{l}{}^{2},\sigma_{\alpha }{}^{2}$, and $\sigma_{\beta }{}^{2}$ represent the variance of l,α, and β, respectively. Range measurement experiments were conducted to determine $\sigma_{l}{}^{2}$ in our previous research [19], from which we learned that $\sigma_{l}{}^{2}$ increases slightly with the measuring distance, and, when the measuring distance is around 2.4 m, the variance of the mean value is about 1.35^2^ mm^2^; for each single measurement point, the variance is about 136.11 mm^2^, which is in accordance with the technical sheet data. In this study, *σ*_l_^2^ = 136.11 mm^2^ is considered invariant. According to the specifications of the LMS100 laser radar and the PANASONIC servomotor, the other two variances can be determined as *σ_α_*^2^ = (1.74 × 10^−30^)^2^ and *σ_β_*^2^ = (3.6 × 10^−50^)^2^. Thus, the covariance matrix $\sum_{l,\alpha ,\beta }$ is obtained.

However, uncertainty in the real Cartesian 3D space appears as the propagation of uncertainty of the raw data in the laser radar scanning space. Since the mapping relationship is non-linear, the covariance matrix $\sum_{x,y,z}$ should be obtained through Equation (6) by performing a linear approximation of the mapping function using a Jacobian matrix, as shown in Equation (7).

(6) $$J(l,\alpha ,\beta )=\left[\begin{array}{l} \frac{\partial x}{\partial l}\;\frac{\partial x}{\partial \alpha }\;\frac{\partial x}{\partial \beta }\\ \frac{\partial y}{\partial l}\;\frac{\partial y}{\partial \alpha }\;\frac{\partial y}{\partial \beta }\\ \frac{\partial z}{\partial l}\;\frac{\partial z}{\partial \alpha }\;\frac{\partial z}{\partial \beta } \end{array}\right]=\left[\begin{array}{ccc} \begin{array}{l} \sin\alpha \cos\beta \\ \cos\alpha \\ \sin\alpha \sin\beta \end{array} & \begin{array}{l} l\cos\alpha \cos\beta \\ -l\sin\alpha \\ l\cos\alpha \sin\beta \end{array} & \begin{array}{l} -(l\sin\alpha +p)\sin\beta \\ 0\\ (l\sin\alpha +p)\cos\beta \end{array} \end{array}\right]$$

(7) $$\sum_{x,y,z}=J\sum_{l,\alpha ,\beta }J^{T}$$

**Figure 3:**
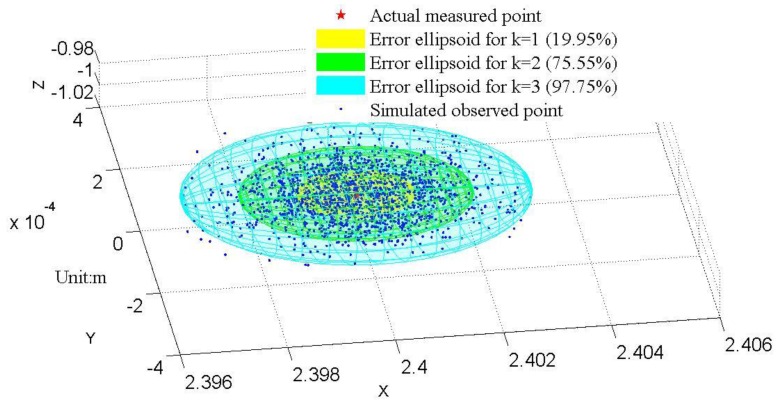
2000 simulated observation points and error ellipsoids for $r^{T}\sum_{x,y,z}{}^{-1}r=k^{2}$.

Thus, the single-point error ellipsoid model for the LRMS is established. Through Equation (4), error ellipsoid for any point in the measurable space can be drawn. As shown in Figure 3, 2000 simulation experiments were conducted to demonstrate the possible observed positions (blue dots) for a given actual point (red star) under the abovementioned uncertainties (*σ*_l_^2^ = 136.11 mm^2^, *σ_α_* = 1.74 × 10^−30^, and *σ_β_* = 3.6 × 10^−50^) by using a normal distribution. Meantime, three error ellipsoids for $r^{T}\sum_{x,y,z}{}^{-1}r=k^{2}$ corresponding to different *k* values are drawn. From the figure, it can be seen that 97.75% of the random simulation results are contained in the error ellipsoid of *k* = 3. Moreover, the frequency of the occurrence of the blue dots becomes higher when they get closer to the actual point, exactly following the rule of 3D normal distribution. Therefore, Figure 3 justifies the use of the single-point error ellipsoid model for evaluating the measurement uncertainty of the LRMS.

## 3. Error Ellipsoid Model for Diameter Measurement of Cylindroid Component

The LRMS was originally designed for diameter measurement of large-scale hot forgings, which are normally cylinders. Hence, it is necessary to assess the measurement uncertainty given the random error of all points. One popular way to detect a cylindroid model from a set of point cloud data is the RANSAC algorithm [21]. The essence of this algorithm is to find the optimal desired model, which can fit the majority of points, thus making it a less error-prone and more robust model-fitting algorithm compared to traditional approaches such as the least squares method. Inspired by this principle, “critical point” theory is proposed, and a mathematical uncertainty evaluation model for the diameter measurement of a cylindroid component based on the single-point error ellipsoid model we just derived is established.

### 3.1. Critical Point on the Cylindrical Surface

**Figure 4:**
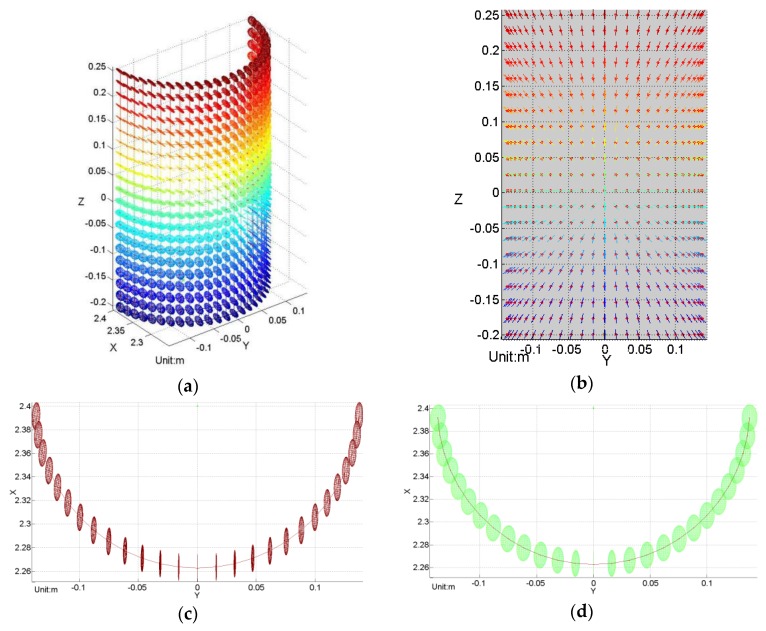
(**a**) Uncertainty ellipsoid map of a standing cylinder surface. (**b**) Front view of *y*-*z* plane direction, (**c**) Top view of the top section of the cylinder. (**d**) Top view of the middle section of the cylinder. The optical center of the laser radar is located at the origin of the coordinate system. (**a,b**) illustrate the distribution of uncertainty ellipsoids on a standing cylinder surface, showing that the range measurement uncertainty is the main error contribution. (**c,d**) imply that the uncertainty in horizontal direction of each point increases towards the cylinder edges. Moreover, the sizes of ellipsoids increase with the range distance and the orientation angle relative to the optical axis, which is along the intersection line of the central scanning plane and the rotation plane.

**Figure 5:**
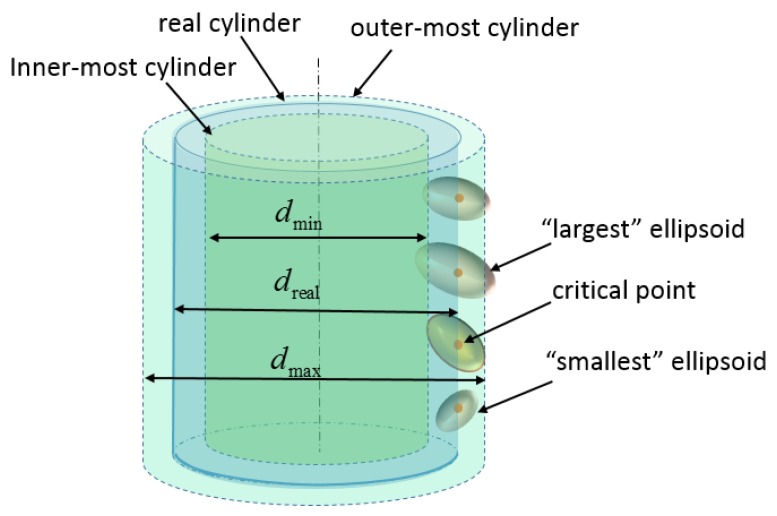
Critical point on a cylinder surface.

Figure 4 gives a straightforward visualization of the distribution of error ellipsoids spread all over the cylindrical surface. It shows a map of 3D error ellipsoids for all sampled points on the surface. The simulation is run under real conditions with the origin of coordinates as the presumed optical center. The color variation of ellipsoids indicates the position change in the z-direction. From the front view, Figure 4b, it is clear that the orientation of the error ellipsoids change with their positions, and the range measurement uncertainties contribute the most; from the top view, in Figure 4c,d, it can be clearly seen that the volumes of error ellipsoids change with their positions in both the horizontal and the vertical direction. According to the principle of the RANSAC algorithm, the cylinder model detected should fit the most points within a certain small-distance threshold. Therefore, it is safe to conclude that the detected cylinder model should intersect most of the error ellipsoids. If two boundaries are set for the possible position of the detected model, as shown by the inner-most and outer-most presumed cylinders in Figure 5, then it can be assumed that diameters of the two boundary cylinders $d_{\min}$ and $d_{\max}$ depend on some certain ellipsoid, which is just tangent to the presumed boundary cylinders. The point that has such a determinant error ellipsoid is defined as the “critical point.” Once the critical point is determined, $d_{\min}$ and $d_{\max}$ are available.

### 3.2. Uncertainty Evaluation Model for a Given Critical Point

**Figure 6:**
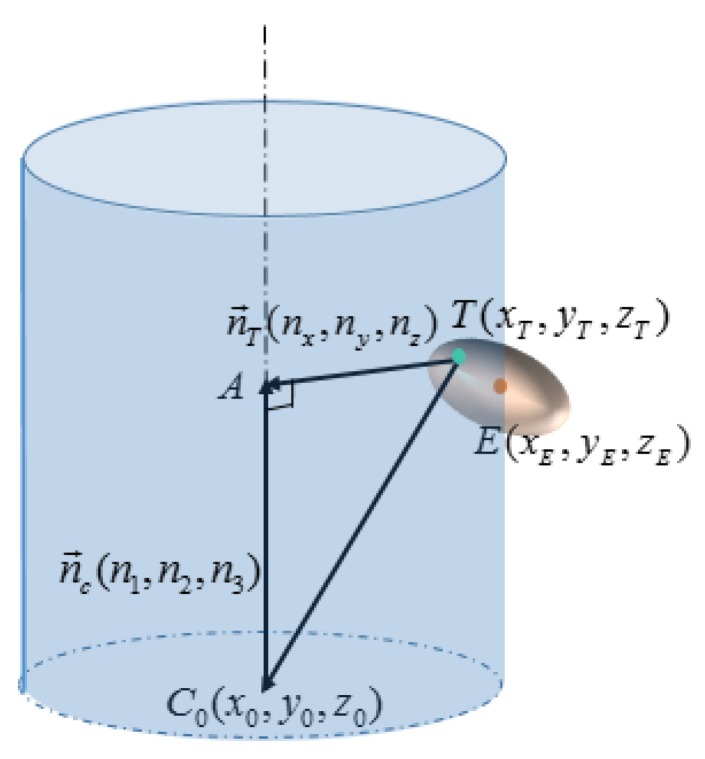
Uncertainty evaluation for a given critical point.

As is shown in Figure 5, the error ellipsoid of the critical point is tangent to the innermost cylinder of $d_{\min}$. If the distance between the critical point and the tangent point in the radial direction of the real cylinder is defined as the “centripetal radius” of this ellipsoid, denoted as $r_{\text{ce}}$, then $d_{\min}=d_{real}-2r_{ce}$. Due to the centrosymmetric property of ellipsoids, $d_{\max}=d_{real}+2r_{ce}$. In order to compute the corresponding diameters $d_{\min}$ and $d_{\max}$ for a given presumed critical point, a rough estimation of the position and dimension of the cylinder is required. As shown in Figure 6, point $C_{0}(x_{0},y_{0},z_{0})$ is the center of the bottom circle of the real cylinder, and ${\vec{n}}_{c}(n_{1},n_{2},n_{3})$ is the normal vector of the central axis. Suppose that point E is the critical point on the cylindrical surface, then the presumed innermost cylinder of $d_{\min}$ is tangent to the ellipsoid of E at point T. Thus, the distance between point T and the central axis |TA| is just the radius of the innermost cylinder $\left|TA\right|=r_{\min}=d_{\min}/2$.

Since $\vec{C_{0}A}=(x_{A}-x_{0},y_{A}-y_{0},z_{A}-z_{0})=\;\left|\vec{C_{0}A}\right|\vec{n_{c}}$, point A can be expressed as

(8) $$A(x_{A},y_{A},z_{A})=\;(x_{0}+\left|\vec{C_{0}A}\right|n_{1},y_{0}+\left|\vec{C_{0}A}\right|n_{2},z_{0}+\left|\vec{C_{0}A}\right|n_{3})$$

where $\left|\vec{C_{0}A}\right|$ can be calculated as the dot product of $\vec{C_{0}T}$ and $\vec{n_{c}}$.

(9) $$\left|\vec{C_{0}A}\right|=\vec{C_{0}T}•\vec{n_{c}}=n_{1}(x_{T}-x_{0})+n_{2}(y_{T}-y_{0})+n_{3}(z_{T}-z_{0})$$

Notice that $\left|\vec{C_{0}A}\right|$ is an expression with regard to the only unknown variables $T(x_{T},y_{T},z_{T})$. Next, vector $\vec{TA}$ can be written as:

(10) $$\vec{TA}=(x_{A}-x_{T},y_{A}-y_{T},z_{A}-z_{T})=(x_{0}-x_{T}+\left|\vec{C_{0}A}\right|n_{1},y_{0}-y_{T}+\left|\vec{C_{0}A}\right|n_{2},z_{0}-z_{T}+\left|\vec{C_{0}A}\right|n_{3})$$

Naturally, the distance between point T and the central axis |*TA*| can be expressed as:

(11) $$r_{\min}=\left|\vec{TA}\right|=\sqrt{(x_{0}-x_{T}+\left|\vec{C_{0}A}\right|n_{1}{)}^{2}+(y_{0}-y_{T}+\left|\vec{C_{0}A}\right|n_{2}{)}^{2}+(z_{0}-z_{T}+\left|\vec{C_{0}A}\right|n_{3}{)}^{2}}$$

Substitute Equation (9) for $\left|\vec{C_{0}A}\right|$ in Equation (11), and $T(x_{T},y_{T},z_{T})$ becomes the only unknown factor in $r_{\min}$. Clearly, $T(x_{T},y_{T},z_{T})$ is the solution to the following optimization function.

(12) $$\left\{ \begin{array}{l} \min\;r=\sqrt{(x_{0}-x+\left|\vec{C_{0}A}\right|n_{1}{)}^{2}+(y_{0}-y+\left|\vec{C_{0}A}\right|n_{2}{)}^{2}+(z_{0}-z+\left|\vec{C_{0}A}\right|n_{3}{)}^{2}}\\ s.t.\;(x,y,z)\in \{g|g(x,y,z)\leq r^{T}\Sigma^{-1}r-k^{2}\;\}\;\left(r=(x-x_{E},y-y_{E},z-z_{E})\right) \end{array}\right.$$

Using the optimization toolbox in Matlab [22], the above problem can be easily solved. With $T(x_{T},y_{T},z_{T})$ available, $d_{\min}=2r_{\min}$ can be obtained. Because of the centrosymmetric property of ellipsoids, $d_{\max}=2d_{real}-d_{\min}$ is also attainable. Thus, the only problem remaining is how to select the proper critical point.

### 3.3. Critical Point Selection Based on Statistical Analysis

Since the “critical” point plays a critical role in determining the uncertainty interval [$d_{\min},d_{\max}$], the critical point should be properly selected so that the uncertainty evaluation is neither too large nor too small. For all the points on the cylindrical surface, the critical point should be selected according to their centripetal radius. In Figure 5, the “largest” and “smallest” ellipsoids refer to the ellipsoids with the largest and smallest centripetal radius, respectively. If the point with the “largest” ellipsoid is chosen as the critical point, it is not hard to imagine that the interval [$d_{\min},d_{\max}$] might be too large in this extreme case. Likewise, if the one with the “smallest” error ellipsoid is deemed as the critical point, the interval might be too small to contain the diameter value of the possible extracted cylinder model.

**Figure 7:**
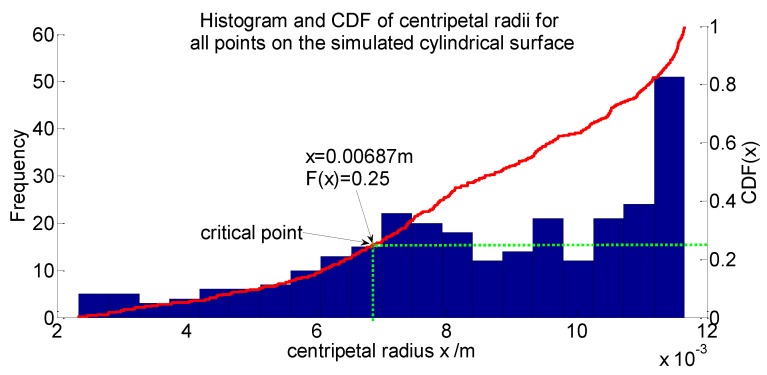
Histogram and cumulative distribution function (CDF) of centripetal radii for all points on the simulated cylindrical surface. (The cylinder is simulated based on the actual experiment condition in Section 4.)

In order to find the proper critical point, statistical analysis of the centripetal radius of all the error ellipsoids on the simulated cylindrical surface can be performed to help address the problem. As shown in Figure 4a, dense packed points are drawn with their error ellipsoids on the cylindrical surface. For every point on the surface, their centripetal radii can be calculated through Equation (12). Statistical analysis of these radii can then be performed by plotting a histogram and a cumulative distribution function (CDF) plot, as shown in Figure 7. From the histogram, it is clear that the points with the “smallest” centripetal radius only possess a relatively small portion of the overall points; as a result, they should not be selected as the critical point, since the cylinder model detected by the RANSAC algorithm would otherwise probably occur beyond the “smallest” centripetal radius range, just as we inferred. However, although the points with the “largest” error ellipsoids possess a major portion, overly large results might be yielded if they are chosen as critical points because the observation points are more likely to occur near the real point. The subsequent experiments can also confirm the two speculations. Therefore, the best option is to choose a middle-centripetal-radius point as the critical point. It is found by simulation that if the centripetal radius at the 25th percentile of the cumulative distribution is used to determine the uncertainty boundary of the cylinder, then over half of the observation points of the simulated points on the cylinder surface would fall within this boundary, since the distance from the observation point to the center of the ellipsoid in the centripetal-radius direction obeys the normal distribution. Further, because the working principle of RANSAC algorithm is to fit the majority of the points, then these 50% points are just enough for the algorithm to fit a cylinder model. Naturally, the fitted cylinder model is among these 50% points. Therefore, the centripetal radius at the 25th percentile of the cumulative distribution can be used to describe the diameter uncertainty appropriately. In Figure 7, the point at which the two green dotted lines meet represents the location of the critical point.

## 4. Experiments and Results

In order to verify the uncertainty model proposed herein, actual and simulated experiments were conducted. The actual experiment was performed by scanning a cylindroid component multiple times and calculating the diameter using the RANSAC algorithm. In the simulation experiment, the uncertainty model was applied for a diameter uncertainty evaluation. Afterwards, the experimental and simulated results were compared and analyzed to justify the validity of the proposed uncertainty model.

### 4.1. Actual Experiment

**Figure 8:**
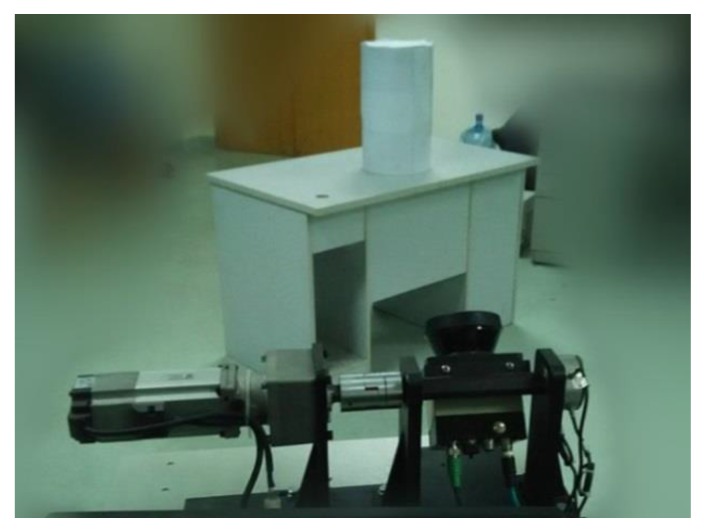
Experimental setup.

**Figure 9:**
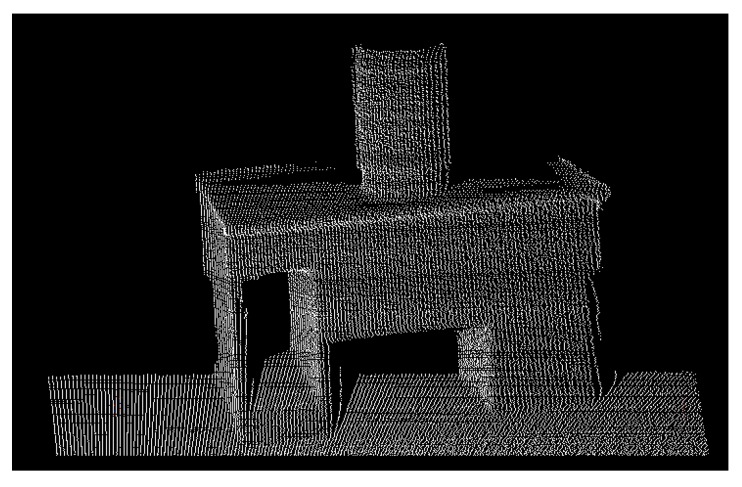
3D point cloud of measured cylinder.

An experiment was conducted to measure the diameter of a standing cylinder. As shown in Figure 8, the measured cylinder was placed on a desk in the middle of the room, nearly 2.5 m away from the radar laser center. In order to test the consistency of the uncertainty result of the evaluation, four groups of measurements were conducted. For each group, the location and orientation of the object were slightly adjusted. To acquire each set of measurements, the object was scanned 10 times. Figure 9 shows an example of a 3D point cloud obtained for the measured cylinder.

To recognize and extract the cylinder part from a large set of point cloud, as is shown in Figure 9, the RANSAC algorithm in the Point Cloud Library (also known as PCL) [23,24] was implemented. The parameters of this detected cylinder are thus obtained, including the normal vector of the central axis, the coordinate of one point on the axis, and the radius value. For each group of experiments, all 10 scanning point cloud datasets were processed to obtain the radius values.

### 4.2. Simulation Based on the Proposed Uncertainty Model

The results of the physical experiment allow a rough estimation of the parameters of the measured cylinder. The proposed uncertainty evaluation model for the diameter measurement can then be used for simulations. For instance, in one set of measurements, the cylinder part is detected with the RANSAC algorithm, and a rough parameter estimation is calculated; by calculating the average of the results from the 10 scans in the experiment, the parameters used in the simulation can be determined, *i.e.*, the axis normal vector ${\vec{n}}_{c}=(0,0,1)$, the radius $r_{\text{real}}=0.138\;m$, and a point on the axis P(0.28,2.53,0.51)    m. Since the plane supporting the cylinder can also be detected using the RANSAC algorithm, an intersection point between the plane and the axis is easy to obtain. The intersection point is the center of the bottom circle of the cylinder $C_{0}(0.28,2.53,-0.1)\;m$. With these parameters, a part of the cylinder surface can be simulated. Five hundred sixty-seven densely packed points are then uniformly selected on the cylindrical surface, and their error ellipsoids are drawn for Equation (4). The selected points represent the simulated scan points, accounting for about 10% of the total cylinder points in the real scan process. The optimization Equation (12) is then solved to acquire all the centripetal radii of these points. Among all these points, the one with the 25th percentile centripetal radius is selected as the critical point. Therefore, the uncertainty interval for the diameter can be evaluated.

### 4.3. Results and Discussion

**Figure 10:**
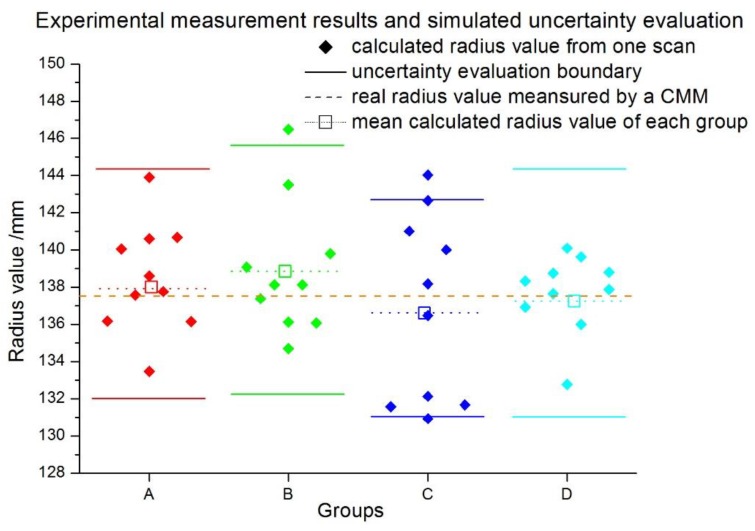
Experimental radius measurement results and simulated uncertainty evaluation results.

To verify the uncertainty model proposed in this study, four actual experiments were conducted for the radius measurement of a standing cylinder. The cylindrical diameter is 137.5 mm, measured by a traditional coordinate measuring machine (CMM). The measuring results are plotted as scatter diamonds of different colors, as shown in Figure 10. Each experiment includes 10 scanning results of the cylindrical diameter, which is displayed as group A, B, C, and D, separately. It is clear that the experimental measurement values vary around the real value with different deviations. The simulation based on the uncertainty model for each group was carried out accordingly. The results [$r_{\min},r_{\max}$] representing the lower and upper boundary were plotted as short straight lines in the graph. From the figure, it can be seen that almost all of the experimental results are distributed in the radius uncertainty interval [$r_{\min},r_{\max}$], except for some extreme outliers. Since the experiment conditions of these four experiments are not the same, the simulated uncertainty intervals are slightly different, which is as expected.

Compared with the existing uncertainty evaluation models, which are mainly focused on the point uncertainty evaluation, the presented approach is extended to geometric features of the cylindrical surface, such as diameter. The traditional uncertainty evaluation are mainly dealing with the single point, whether it is a directly measured point or an indirectly measured point which has an explicit mathematical function of the error propagation and aggregation. The actual uncertainty is calculated by variance computation from the data of multiple measurements according to the explicit error propagation or aggregation expressions. However, for the massive point cloud, no explicit expression exists, such as the abovementioned cylinder diameter case. Using our method, the uncertainty interval is effectively determined as approximately ±6.36 mm in our experiment. This method could be further used for the uncertainty evaluation of geometric features of differently shaped objects in general, such as conic and spherical surfaces. It is especially suitable for the uncertainty evaluation of geometric features of a massive point cloud that is obtained by different scanners, such as the laser ranger, laser tracker, and the articulated arm coordinate measuring machine.

## 5. Conclusions

In this study, we proposed an uncertainty evaluation model for the diameter measurement of a standard cylindroid component based on the error ellipsoid theory. To this end, we first established a single-point error ellipsoid model for the laser-based measurement system. Then, based on this error ellipsoid model, an uncertainty evaluation model and algorithm for cylindrical diameter measurement were proposed. To obtain the evaluation model, the critical point theory was first proposed, inspired by the RANSAC algorithm. Furthermore, statistical analysis of the centripetal radii of all points on the cylindrical surface was performed to determine the optimal critical point. Based on the critical point, the diameter uncertainty interval was obtained. To verify the uncertainty evaluation model, four experiments performing diameter measurement and a simulation using the proposed model were conducted. The experimental results of the uncertainty evaluation consistently matched well with the predicted ones. Therefore, the uncertainty evaluation model and algorithm for cylindrical diameters can provide a reliable method for actual measurements, demonstrating the validity of the proposed model.

In future work, we will investigate methods to further improve the accuracy of this model and try to apply it to other laser-based measurement systems.
